# Reply to Davis, A.K. Monarchs Reared in Winter in California Are Not Large Enough to Be Migrants. Comment on “James et al. First Population Study on Winter Breeding Monarch Butterflies, *Danaus plexippus* (Lepidoptera: Nymphalidae) in the Urban South Bay of San Francisco, California. *Insects* 2021, *12*, 946”

**DOI:** 10.3390/insects13010064

**Published:** 2022-01-06

**Authors:** David G. James, Maria C. Schaefer, Karen Krimmer Easton, Annie Carl

**Affiliations:** 1 Department of Entomology, Washington State University, 24106 North Bunn Road, Prosser, WA 99350, USA; 2 2558 Mardell Way, Mountain View, CA 94043, USA; seasluglover@yahoo.com; 3 435 Marion Avenue, Palo Alto, CA 94306, USA; eastonfamilyfive@gmail.com; 4 2661 Waverley Street, Palo Alto, CA 94306, USA; anniejcarl@hotmail.com

## 1. Introduction

This is a reply to the comment from Davis [1], who commented on our work “First population study on winter breeding monarch butterflies, *Danaus plexippus* (Lepidoptera: Nymphalidae) in the urban south Bay area of San Francisco, California” [2].

Briefly, Davis contested our suggestion that monarchs overwintering as a breeding population in the south Bay area of San Francisco may still contribute to northward and eastward migration during spring. His primary argument was that our tagged monarchs had shorter wings than migratory fall monarchs in the eastern and western US and were similar in size to individuals in non-migratory monarch populations. Here we show that large wings are not a pre-requisite for spring migration in monarch butterflies.

## 2. Background

Davis [1] provides a lengthy summary of the current status of western monarchs within the context of our study. We add the following comments.

While Tropical Milkweed (*Asclepias curassavica* L.) is a common non-native milkweed in San Francisco urban areas, it is not the only non-native species grown in gardens. In the south Bay area, the African milkweeds, *Gomphocarpus physocarpus* E. Mey and *Gomphocarpus fruticosus* L., appear to be as frequently cultivated as *A. curassavica*. Our study was primarily conducted to investigate the winter ecology of breeding monarchs in the south Bay area, not to determine whether winter breeding monarchs contribute to the western population as Davis [1] states. However, from the rearing of ~500 larvae, we did obtain limited data suggesting that the winter breeding population may have contributed to the spring migration.

Davis [1] appears to confuse monarchs moving inland (from the coast to the Sierra Nevada) from traditional overwintering colonies in February–March (not March–April) with the first generation of new adults which appear in inland areas in April–May. Overwintered butterflies fly inland (up to 200 km) during February–March ovipositing on milkweed. These eggs and larvae develop during March–April to produce the first generation. It is this generation that migrates north and east beyond California into the greater west [3]. Overwintered butterflies do not migrate out of California.

We were careful in our study, when drawing the conclusions, to couch them in terms that recognized that our data were insufficient to provide definitive recommendations. Thus, while we presented data and observations indicating the importance of non-native milkweeds to the persistence of winter breeding populations in the south Bay area, we did not go a step further and declare that providing non-native milkweeds for monarchs is an “effective conservation strategy” as Davis [1] states.

## 3. Large Wings Are Not Needed by Spring Migrating Monarchs

Davis [1] presents an alternative interpretation of our data that indicates the monarchs we released in March–April did not leave the south Bay area and become spring migrants to the north and east. Davis bases this on the fact that our reared monarchs averaged a forewing length of 47.8 mm, which is substantially lower than the average (50.8 mm) reported in the literature for migratory monarch populations in the eastern and western US. However, all the data referenced by Davis pertain to fall migrants, not spring migrants. We know very little about the physical and physiological characteristics of the first spring generation of adult monarchs in the eastern or western US. This is the generation that develops in Texas or inland California and migrates during April–May to a higher latitude summer breeding range in both the east and west [3,4]. However, there is one paper that looked at the wing sizes of monarchs seasonally in Minneapolis, Minnesota [5]. Herman showed clearly that first-generation monarchs migrating from the south into MN during May–July had significantly smaller hindwings than pre-migratory or migrant monarchs in August–September. This makes sense, because monarchs migrating north in the spring display a different flight strategy than monarchs migrating south in the fall. Fall migrants use a soaring and gliding strategy [6] extensively, which clearly benefits from large wings with a large surface area. In contrast, observations on spring migrants suggest that they largely use powered flight relatively close to the ground [O. R Taylor, pers. comm], allowing them to cover an average of 76.1 km/day and sometimes more than 100 km/day [7]. This is more than double the average daily pace of the southward fall migration [8]. This powered flight strategy would not benefit from large wings, and spring migrants do not have large wings, as shown by Herman [3]. Thus, the alternative interpretation of winter breeding monarchs and their progeny in the Bay area being physiologically unable to effectively migrate, as posited by Davis [1], is not credible. Monarchs migrating northward from the Bay area in spring would be expected to have shorter forewings than fall migrants.

We now have data on forewing lengths of another cohort of monarchs reared in the same south Bay location, this time during fall (September–November 2021). These butterflies had a mean forewing length of 51.2 mm (n = 467), comparable to the average forewing length of fall migrants reported by Davis [1] (50.8 mm). Thus, monarchs, captive-reared under fall conditions in the Bay area attained the expected size of fall migrants despite being reared on the non-native milkweeds, *G. physocarpus* and *A. curassavica.*

## 4. The Importance of Physiology in Determining Migration Status in Monarchs 

Statements provided by Davis [1] appear to indicate confusion about the importance of individual and cohort physiology in determining the reproductive and migration status of monarchs. For example, in his synopsis and in the third paragraph of his “alternative interpretation” section, he talks about the offspring of migrants retaining “migratory nature”. Migration and reproductive status are not traits that are passed from one generation to the next. Reproductive dormancy and migration in late-summer and fall monarchs are induced during larval [9] and/or early adult stages [10] by declining daylengths and cooling temperatures. Sun angle at solar noon (SASN) also appears to play a role, with most migration occurring when SASN is between 46 and 57 degrees [11]. The environmental cues necessary for spring migration have not been characterized, but they are likely to be increasing daylengths, increasing SASN and warming temperatures. Consequently, developing individual monarchs and populations exposed to inductive environmental conditions will largely respond accordingly. There is no reason to think that the induction of migratory behavior will not occur during monarch development in the Bay area and other parts of California in spring, to produce adults programmed to migrate.

Despite the lack of study on developing and adult first-generation monarchs in the eastern or western US during April–May, we do have a single data point showing that a monarch, progeny of a continuously breeding laboratory population but exposed during larval development (in a glasshouse) to warming temperatures and increasing daylengths and SASN in April and early May, became a migrant. “Big Boy” was one of six radio-tagged monarchs released in eastern Kansas in mid-May 2010 for National Geographic by Chip Taylor and Martin Wikelski (https://www.youtube.com/watch?v=I0kIHlu-ikM, accessed on 20 December 2021). Tracked electronically, Big Boy flew 11.4 miles NE, indicating that spring daylengths, SASN and temperatures during development produced a migratory monarch (Taylor, pers comm.).

In the west, a single monarch captured and tagged on 24 May 2015, while migrating north along the Trinity River in northern California, was recovered 5 weeks later, 707 km ENE, in Twin Falls, Idaho [3]. This individual, progeny of an overwintered female that developed in California during April–May clearly was migratory.

## 5. Is It Possible for Less than 2000 Monarchs to Produce a Population of 200,000 Monarchs in One Season? 

From an overwintering population of less than 2000 monarchs at 249 coastal California sites in November 2020, the western monarch population increased 100-fold in 2021 to more than 200,000 (http://www.westernmonarchcount.org, accessed on 20 December 2021). In the 2020/2021 New Year count there were only 1039 butterflies at 149 overwintering sites. It is unlikely that the increase seen in 2021 can be accounted for by the success of this tiny overwintered population alone. If there were little more than 1000 butterflies at the end of overwintering there would have been around 500 females with a lesser number surviving to lay eggs. In our paper [2], we speculated that the progeny of winter breeding monarchs in the San Francisco Bay area may contribute to spring migration just like the progeny of monarchs that overwinter at traditional sites [12]. It is also possible that winter and spring breeding populations in urban areas as far south as San Diego (latitude 32.42 N) may also contribute to spring migration. In the eastern US, Knight et al. 1999 [13] showed that first-generation monarchs developing in March–April, 20 miles SE of Gainesville, FL (29.32 N)—a latitude similar to that of San Diego—joined the spring migration. While it is impossible to know the size of the winter breeding population that occurred in the urban areas of California in 2020/2021, we considered it possible that it may have been much larger than the total number of monarchs (ca. 1900) counted at traditional overwintering sites in 2020 [2]. Consequently, we suggested that there is good reason to believe that the winter breeding population contributed to the substantial increase seen in the western population in 2021. Other factors may have also played a role in the increase, including the “leakage” of spring migrants westward into AZ from the dispersing Mexican overwintering colonies [14].

## 6. Conclusions

In summary, we disagree with the assertion by A. K. Davis that our late winter–early spring reared monarchs did not have wings large enough to be spring migrants [1]. On the contrary, they were appropriately sized for spring migration. We base this on published research showing that spring migrants in the eastern US have smaller wings than fall migrants [5] and the fact that spring migrants utilize a powered, lower-to-the-ground flight strategy than large-winged fall migrants, which soar and glide [6]. We also contest the idea that the offspring of resident (non-migratory) monarchs cannot contribute to the spring migration. Research on the association between environmental cues (primarily changes in daylength, SASN and temperature) and monarch physiology clearly shows that the reproductive and migratory behavior of newly eclosed adult monarchs is a function of environmental cues experienced during immature and/or teneral development [9,10,11]. Thus, it is reasonable to conclude that monarchs from winter breeding populations developing during spring, when daylengths and SASN are increasing and temperatures rising, will be migratory shortly after eclosion and contribute to the late spring migration of California monarchs to the greater west. Further, we also think it is reasonable to suggest that the uncountable thousands of winter breeding monarchs in near-coastal urban areas of California in 2020/2021 played at least some role in the current astonishing rebound of the western monarch population. We do agree with the final sentence in the comment by Davis [1], namely that the conservation value of urban sites in California with non-native milkweeds has not been resolved by our study. Rather, it is an initial study that highlights factors like non-native host plants, pathogen levels and potential spring migration, that need more detailed and focused research.
